# Teaching presence predicts cognitive presence in blended learning during COVID-19: The chain mediating role of social presence and sense of community

**DOI:** 10.3389/fpsyg.2022.950687

**Published:** 2022-08-29

**Authors:** Ling Li

**Affiliations:** College of Foreign Languages, Zhejiang Normal University, Jinhua, China

**Keywords:** teaching presence, social presence, sense of community, cognitive presence, blended learning, COVID-19

## Abstract

With the continuous lockdown and staying home strategies of COVID-19, both instructors and learners have met with the presence challenges in language learning. To address the complex and dynamic relationships of different presences in blended learning during COVID-19, based on the Community of Inquiry framework, 215 Chinese English learners were obtained as samples for an empirical test. SPSS 23 and PROCESS for SPSS were utilized to examine the hypotheses. Results indicate that teaching presence (TP) has a significant direct positive impact on social presence (SP), sense of community (SoC), and cognitive presence (CP). SP has a significant positive impact on CP and partially mediates the relationship between TP and CP. SoC is also found to impact CP and partially mediates the relationship between TP and CP. The findings also validate the chain mediating role of SP and SoC between TP and CP. Pedagogical implications are discussed.

## Introduction

During the period of COVID-19, instructors were forced to creatively reconceptualize education practice and the education of future generations, among which higher education institutions acted as the first to shift to learning online (Pokhrel and Chhetri, 2021). In China, under the policy of “Suspending Classes without Stopping Learning” (Zhang et al., 2020), universities flexibly selected teaching and learning modes depending on the changing situation of the COVID-19 pandemic. Another challenge is the reopening of schools and universities after social distancing and restrictive movement policies. Most universities replaced face-to-face learning with online learning since March 2020, and chose blended learning indefinitely (Chamberlain et al., 2020) after a smooth transition to normal offline education. Some other studies in different fields across the world also rate blended learning higher compared to face-to-face traditional learning or fully online learning (Amir et al., 2020; AlQhtani et al., 2021; Amro, 2022), but results in Spain (Alonso-García et al., 2021) and Philippines (Baloran, 2020) show a preference for face-to-face learning rather than online-blended learning. Besides the technological support and teacher inexperience of this remote teaching mode, teacher virtual presence and learner engagement are important factors that affect the learning process (Christopher et al., 2020; Matthews et al., 2021).

With no exception, foreign language (FL) teachers adapted to the restrictions imposed across the globe by moving to crisis-prompted remote language teaching (Gacs et al., 2020). Owing to the social distancing actions taken in the quarantine period, many online platforms (e.g., Blackboard, Zoom, and Instagram) were utilized for educational purposes (Tofade and Daftary, 2020) across the world. In China, WeChat, DingTalk, QQ, and some other online learning management systems were frequented by thousands of learners since the outbreak of the epidemic (Yang, 2020). Nevertheless, researches show that the willingness of students in choosing online learning is low and most students are not skilled at online learning in China (Bao, 2020; Zhang et al., 2020). The downside of long-term distancing and online isolation may directly impact learner engagement and effectiveness in general (Carlson, 2020), as evidenced in the UK (Walters et al., 2021), Australia (Abdel Latif, 2022), Qatar (Abouhashem et al., 2021), Malaysia (Al-Kumaim et al., 2021), South Korea (Almusharraf and Bailey, 2021), Spain (Alonso-García et al., 2021), Saudi Arabia (Alqahtani et al., 2021), India (Basri et al., 2022), and so on. But online language learning during the no-direct contact period was not an option, but a necessity (Gacs et al., 2020).

FL teaching at university did not stay untouched by the severe disruption. Different from planned online language teaching, crisis-prompted remote language teaching might suffer from insufficient training, limited accessible digital resources, increasing workload for instructors, and pandemic trauma or disconnectedness (Gacs et al., 2020). Thus, the community is the key concept to rethinking how the traditional classroom operates successfully in online or blended environments. Building up a learning community has a positive effect on students' learning and helps increase student engagement and motivation, as reported by Fiock (2020). Lomicka (2020) stresses the significance of SPs in engaging learners during the process of establishing a virtual language learning community. Learners' perception of SP from affective, interactive, and cohesive sub-dimensions can help to identify possible obstacles and accelerating factors in maintaining a sense of connectedness in a virtual classroom. Complex as language learning is, the micro-level of social interaction and the meso-level of sociocultural communities remain very vital to the process (The Douglas Fir Group, 2016). Based on socio-cultural language teaching pedagogies, interaction is vital to knowledge negotiation and authentic language exercise. A predictive relationship is also found between the linguistic characteristics of discussion and student learning performance (Yoo and Kim, 2014).

The Community of Inquiry (CoI) model, comprising TP, SP, and CP, is one of the most influential and informative research frameworks arising from social constructivism in online or blended learning contexts (Kozan and Caskurlu, 2018). According to Garrison and Akyol (2013), it is through the collaboration and interaction of a group of individuals that personal meaning and mutual understanding can be constructed. The three presences of the CoI model, when united, contribute greatly to learner satisfaction and perceived learning (Mills et al., 2016), and online learning success is closely related to a sense of online learning community to a certain degree (Sun and Chen, 2016). Literature shows that blended or hybrid learning environments are superior, though slightly, than fully online environments in developing or sustaining one or more presences (Akyol et al., 2009; Shea and Bidjerano, 2013; Traver et al., 2014); the relationship between the three presences and other variables (e.g., SoC), however, is not fully explored after the transition from COVID-19 remote teaching to normal blended learning. Thus, this study intends to fill this void.

Despite the varying definitions of presence in online or blended learning environments, a tacit consensus was reached that the presence of both instructors and learners contributed to the shaping of a learning community, especially toward the establishment of shared learning goals (Jiang and Koo, 2020; Wang and Liu, 2020; Kim et al., 2021; Roque-Hernández et al., 2021). Without the virtual TP and SP, home-bound students might be bored or feel isolated (Qiu et al., 2020; Abouhashem et al., 2021; Naidoo et al., 2022) due to the lockdown and lack of a conducive learning environment. Due to many uncertainties of instructor competence during the COVID-19 period and learner socio-emotional characteristics (Kast et al., 2021), it is essential to evaluate TP from the angle of learners. Learners' self-reported data about TP is another perspective reflecting instructors' design and organization of learning materials, facilitation, and direct instruction in the language teaching process. A high level of SP is closely related to learner participation in the learning environment and active interaction with course participants toward shared learning goals (Hilliard and Stewart, 2019), thus facilitating creative thinking and deep continuous learning (Garrison and Akyol, 2013). It is necessary to include SoC in the study for a detailed description of learners' feelings about the blended learning community. As Rovai (2002a) describes SoC as a construct composed of connectedness and learning, it is reasonable to put SoC as the mediator connecting SP and CP.

We are interested in the socio-emotional aspects of learners in blended learning during the pandemic period, especially learners' perceptions of TP, SP, and CP under the CoI framework, as well as learners' SoC. Previous studies were conducted on the relationship of the three presences in blended learning. However, the literature did not take into consideration the context of the COVID-19 pandemic as well as the joint effect of learners' perceived SP and SoC. Thus, this research offers a possibility to examine the collective effect of learners' perceived SP and SoC on the association between TP and CP.

### Literature review and hypotheses

With a social-constructivist theoretical basis (Garrison and Akyol, 2013), the CoI model focuses on the learning process first and foremost (Akyol and Garrison, 2011). Adopting the definition and 34-item instrumental scale of the original CoI model (Arbaugh et al., 2008), TP refers to “the design, facilitation, and direction of cognitive and social processes for the purpose of realizing personally meaningful and educationally worthwhile learning outcomes” (Anderson et al., 2001; p. 5). SP is described as “the ability of participants to identify with the community (e.g., course of study), communicate purposefully in a trusting environment, and develop inter-personal relationships by way of projecting their individual personalities” (Garrison, 2009; p. 352). CP is defined as “the extent to which the participants in any particular configuration of a community of inquiry are able to construct meaning through sustained communication” (Garrison et al., 2000; p. 89).

The relationships among the three presences in the original CoI model have been explored by many previous studies. It has been found that TP and SP were perceived to influence CP (Shea and Bidjerano, 2009; Garrison et al., 2010), which was corroborated by the results of Archibald (2010). Ke (2010) adopted the concepts of three presences, yet with different operationalized definitions, and concluded that TP played a significant role in promoting the development of SP and CP. Another study also reported that the three presences were interrelated with each other using the methods of Spearman's rank correlation and partial correlation (Kozan and Richardson, 2014), but a different result was that CP seemed to carry the potential of influencing the relationship between TP and SP, while a more recent study (Gutiérrez-Santiuste et al., 2015) has shown that SP and TP both affected CP, but SP proved to have a higher effect using multiple regression analysis. Furthermore, some other research made a deeper analysis of the associations among the three presences, for example, the relationship between the subdimension of SP (affective, interactive, and cohesive) and CP (triggering event, exploration, integration, and exploration) has been investigated using social network analysis (Rolim et al., 2019), in which results indicated that the affective subdimension of SP had a stronger correlation with the integration and resolution subdimensions of CP, while the interactive subdimension was strongly related with triggering events and exploration stage. Another meticulous research has focused on the developmental process of sub-categories of TP (design and organization, facilitation, and direct instruction) and its influence on students' learning (Wang and Liu, 2020), in which the findings reported that design, organization, and facilitation benefited students' cognitive learning and interaction, while direct instruction might go beyond the line and hinder students' knowledge construction.

Some other studies revised the COI model by adding some new variables into the COI model and investigating the relationship between these new variables and the original three COI presences. For example, a fourth presence named learning presence was proposed (Shea et al., 2010, 2014), followed by the exploration of the mediating role of learning presence in the context of China (Ma et al., 2017), emotional presence by Cleveland-innes and Campbell (2012) and Stenbom et al. (2016), instructor presence by Richardson et al. (2015), and instructor SP (Richardson et al., 2016).

Similar to this study, some previous studies explored the relationship between CoI presences and some new variables but did not modify the original CoI framework. For example, Akyol and Garrison (2008) identified significant correlations among the three presences, learning motivation, and learner expectation by analyzing online postings. Another study investigated the mediating roles of the three presences with learning performance as the dependent variable in a blended learning context (Law et al., 2019), and the results showed that TP affects SP and CP directly. As reported elsewhere, self-efficacy was investigated as a mediator in the relationship between TP, SP, and CP (Lin et al., 2015), while gender, age, and academic level also might play mediating roles in impacting students' learning experiences (Shea and Bidjerano, 2013).

### TP and CP

Positive correlations among TP, SP, and CP have been found in previous research (Garrison et al., 2010; Ma et al., 2017; Dempsey and Zhang, 2019). TP has been reported to play a significant role in predicting CP (Joo et al., 2011; Hilliard and Stewart, 2019) as it could promote critical thinking (Garrison and Akyol, 2013) and enhance online learning via social networking sites (Lin et al., 2015). In short, TP is closely related to both learning experiences and actual learning outcomes (Li et al., 2021), serving as a bridge between SP and CP (Miller et al., 2014; Rockinson-Szapkiw et al., 2016). To help students undergo a natural transition from SP to CP, the TP of a facilitator is a necessity (Joksimović et al., 2015). It has been discovered that students reacquainted themselves through SP and oriented themselves to the cognitive assignment through TP (Stein et al., 2007). According to Garrison and Cleveland-Innes (2005), TP impacts CP through course structure design and instructor facilitation of the learning process.

However, other studies also argued that TP had no impact on SP in terms of feedback support analysis (Borup et al., 2015), and it should be monitored more carefully to a certain extent for the facilitation and promotion of student knowledge construction (Zhao et al., 2014; Wang and Liu, 2020). As too much TP might dominate peer interaction and hinders learners' initiatives for participation, resulting in decreased, scattered, and unrelated responses. In this case, students should be encouraged and facilitated to manage their own learning toward higher-order thinking, and teachers should step out of the centrality by lessening direct instruction to promote students' knowledge negotiation (Wang and Liu, 2020). We argue in this study, that with remote teaching experiences in 2020 and comparative well-planned structures in 2021, students' perceived TP will have a positive significant influence on CP in blended learning during COVID-19.

**Hypothesis 1 (H1)**. *TP has a positive and significant contribution to CP in blended learning during COVID-19*.

### SP between TP and CP

It has been validated that CP was supported and sustained by SP through critical thinking among a community of learners (Rourke et al., 1999). According to Shea and Bidjerano (2009, 2012), students' perceived TP and SP predict 70% of the change in CP with learning presence as the mediator. Researchers also explored the causal relationships among TP, SP, and CP (Garrison et al., 2010), strengthening the vital role of TP in determining SP and CP. Another research found that when self-regulated learning readiness and prior online and collaborative learning experience are controlled, 69% of the variance in the students' level of CP could be explained by teaching and SP (Archibald, 2010). Szeto (2015) also found out that TP played a more determining role in achieving the planned learning outcomes than SP and CP in a blended synchronous engineer learning and teaching mode, but another study suggested a higher effect of SP on CP (Gutiérrez-Santiuste et al., 2015). Therefore, CP is strongly correlated with teaching and SPs (Ke, 2010; Joksimović et al., 2015; Tirado Morueta et al., 2016; Ma et al., 2017), and the partial mediating role of SP between TP and CP was significantly validated by many studies (Garrison et al., 2010; Joo et al., 2011; Kozan and Richardson, 2014). Thus, hypotheses 2 to 4 were developed as follows.

**Hypothesis 2 (H2)**. *TP has a positive and significant relationship with SP in blended learning during COVID-19*.

**Hypothesis 3 (H3)**. *SP has a positive and significant relationship with CP in blended learning during COVID-19*.

**Hypothesis 4 (H4)**. *SP plays a positive and significant mediating role in the relationship between TP and CP in blended learning during COVID-19*.

### SoC between TP and CP

There is a significant association between learners' SoC and perceived TP (e.g., design and facilitation), but more research is necessary to explore deeply the differences in blended and online learning environments and the reasons (Shea et al., 2006). Another study reports that the inclusion of an asynchronous audio element strengthens students' SoC and the instructor's projection of immediacy, providing a more individualized communication with students (Ice et al., 2007). When students feel that the instructor cares more about them (TP), they end up with increased retention of content (CP). It is important for instructors to provide timely announcements, building up a strong sense of connection between students and the instructor (d'Alessio et al., 2019). Instructors' immediacy and prompt reply (TP) seem to be vital in sustaining the development of a community of inquiry (Oliphant and Branch-Mueller, 2016).

Rovai (2002b); reported that learners' SoC affected their perceived learning, as reported the same in other studies, whether online (Trespalacios and Perkins, 2016) or web-based (Overbaugh and Lin, 2006), and the role of perceived learning to predict final course grades is validated in an educational community of inquiry (Rockinson-Szapkiw et al., 2016), and those with higher SoC may result in higher learning satisfaction and knowledge sharing as well (Chen and Chiou, 2014; Guo and Cheng, 2016; Yapici, 2016). Furthermore, there is a slight positive relationship between SoC and academic achievement (Wighting et al., 2009), with standardized exams being the measurement of academic achievement. However, Overbaugh and Nickel (2011) argued that an SoC was not necessary for knowledge sharing considering the time and energy needed in the community-building process. We argue that students' SoC during the COVID-19 period is important in blended learning. Therefore, hypotheses 5 to 7 are formed as follows.

**Hypothesis 5 (H5)**. *TP has a positive and significant relationship with SoC in blended learning during COVID-19*.

**Hypothesis 6 (H6)**. *SoC has a positive and significant relationship with CP in blended learning during COVID-19*.

**Hypothesis 7 (H7)**. *SoC plays a positive and significant mediating role in the relationship between TP and CP in blended learning during COVID-19*.

### SP and SoC

Tu and McIsaac (2002) analyzed three sub-dimensions of SP (technological attributes, learners' personal perceptions, and strategies of engaging learners in interactions) and found that the three sub-dimensions were vital to establishing an SoC among learners. Lomicka and Lord (2007) reported that the projecting of SP characteristics helped to establish the respective communities and to strengthen feelings of rapport. Moreover, SP can promote an SoC and a feeling of positive interpersonal dynamics in a blended course of teacher education (Remesal and Colomina, 2013). Lomicka (2020) also claimed that cultivating SP among language learners was very important to maintain a sense of connectivity in the virtual classroom. In short, SP plays a crucial role in influencing the sense of a classroom community (Wang et al., 2021). Thus, hypotheses 8 and 9 are formed.

**Hypothesis 8 (H8)**. *SP has a positive and significant relationship with SoC in blended learning during COVID-19*.

**Hypothesis 9 (H9)**. *SP and SoC play a positive and significant chain mediating role in the relationship between TP and CP in blended learning during COVID-19*.

## Methodology

### Participants

In this study, based on convenience sampling, 215 freshmen students (41 male students, accounting for 19%, ranging from 18 to 20 years of age) having two college English courses at the Zhejiang Yuexiu University of Foreign Languages (ZYUFL) in Eastern China took part in this current research. Noticeably, non-English major students in ZYUFL are required to take college English as a compulsory course for at least 2 years, exclusively utilizing English as the medium for instruction and learning. ZYUFL promoted blended learning in 2018 with financial support from the University Board. With about 2,500 freshmen students, ZYUFL had eight classes of 350 students (about 14%) practicing blended learning mode. Participants in this study were recruited on the basis that they had blended English learning experience for at least 1 year.

### Procedure

There were two steps in this study. For step one, at the very beginning of October 2021, students of eight classes practiced the blended learning model, comprising 4 h in the face-to-face classroom and 1 h online. For face-to-face learning, assignments included lectures, discussions, and project learning. For online learning, video watching, passage reading, asynchronous text discussion, and online quizzes were the main tasks. For step two, at the end of the semester, questionnaires were presented in online links by instructors during class break time. Students were informed of the research purpose and were encouraged to take part in the survey via phone voluntarily (the three questionnaires concerning TP, SP, SoC, and CP were organized consecutively in a single link as a composite questionnaire), which was conducted anonymously and would not affect their final grade. Necessary guidance and help were provided by the instructor in terms of filling out the questionnaire, and informed consent of the students to take part in the current study was also gained. With a response rate of 61.4%, the study collected 215 valid samples for subsequent data analysis.

### Measures

All items in the questionnaire were measured on a 5-point Likert scale ranging from 1 (strongly disagree) to 5 (strongly agree). A high score in each item indicates a high level of perception of the corresponding variable among the students.

### TP (teaching presence)

The TP questionnaire, validated from previous studies in online and blended learning environments (Akyol et al., 2009), was utilized to evaluate students' perceptions of TP in their blended English learning environment. TP was measured from three sub-dimensions: design and organization (four items, e.g., the teacher clearly communicated important course topics), facilitation (six items, e.g., the teacher encouraged my classmates to explore new concepts in this course), and direct instruction (three items, e.g., the teacher provided feedback in a timely fashion). The same scale was utilized by many researchers, and the reliability and validity were high in the Chinese context (Ma et al., 2017). In this study, Cronbach's α coefficient is 0.990 and KMO value is 0.958.

### SP (social presence)

The SP questionnaire was adopted from Arbaugh et al. (2008). The questionnaire consisted of nine items: affective expression (three items, e.g., I was able to form distinct impressions of some classmates), open communication (three items, e.g., I felt comfortable participating in the course discussions), and group cohesion (three items, e.g., I felt comfortable disagreeing with my classmates while still maintaining a sense of trust). Confirmed reliability and validity have been validated in different research works (Garrison et al., 2010; Kozan and Richardson, 2014; Manwaring et al., 2017) and especially with Chinese students as the samples (Ma et al., 2017). The reliability and validity are 0.936 and 0.920, respectively.

### CP (cognitive presence)

This study treated CP as the dependent variable based on previous literature (Garrison et al., 2010; Shea et al., 2010; Kozan and Richardson, 2014), which was measured on a 12-item scale with four sub-dimensions: triggering events (three items, e.g., Problems posed increased my interest in course issues), exploration (three items, e.g., I utilized a variety of information sources to explore problems posed in this course), integration (three items, e.g., Combining new information helped me answer questions raised in course activities), and resolution (three items, e.g., I can describe ways to test and apply the knowledge created in this course). In the Chinese context, previous research has shown high reliability and validity (Ma et al., 2017). The reliability is 0.984 and KMO value is 0.959.

### SoC (sense of community)

The SoC was measured by a scale revised by Rovai and Jordan (2004) with two sub-dimensions: connectedness (five items, e.g., A sampling item is “I felt that students in this course cared about each other”) and learning (five items, e.g., I felt that I was encouraged to ask questions). The scale was validated by many previous studies (Overbaugh and Lin, 2006; Shackelford and Maxwell, 2012; Oliphant and Branch-Mueller, 2016; Shea, 2019; Pelletier et al., 2021). Reliability and validity have been validated in a study conducted in Taiwan (Chen and Chiou, 2014). In this study, the reliability is indicated by Cronbach's α value = 0.930 and the KMO value is 0.863.

### Method of data analysis

At first, SPSS23.0 was used to test the reliability and validity of each variable (i.e., TP, SP, CP, and SoC). Then, PROCESS v3.3 for SPSS as the statistical package was used to conduct a regression analysis, and the structural equation model was tested with path analysis. The study adopted the 5,000 re-sample bootstrapping analysis to examine the indirect effect. Multiple regression analysis was substantiated to test the corresponding indirect effect via PROCESS v3.3 for SPSS.

## Results

### Preliminary analysis of data

Students' perceived level of TP in blended learning during the pandemic has been high with a total mean score of 4.04 (SD = 0.93), while the total mean score of SP in blended learning during the pandemic was 3.68 (SD = 0.79), of SoC was 3.78 (SD = 3.78), and of CP was 3.73 (SD = 0.88). Students had a higher mean score of TP compared to that of SP, SoC, and CP. The results indicated that students rated TP comparatively high, which might be due to instructors' continuous effort in course design and facilitation both online and offline.

Before the assessment of the mediation model, a preliminary analysis of the data was run to evaluate the possibility of running the mediation analysis. According to Baron and Kenny (1986), it is a prerequisite to test the relationship between the independent variable and the dependent variable for running the mediation analysis. In this study, the preliminary data analysis began with the test of whether there was a significant association between TP and CP. Therefore, the means, standard deviation, and correlation among variables were calculated and Table 1 showed the results. The correlation analysis run by SPSS 23.0 revealed a positive correlation between TP and CP (r = 0.745, *p* < 0.01), which corresponded to our hypothesis. It provided preliminary evidence that supported the continuing analysis of the hypothesis testing.

**Table 1:** Descriptive statistics and Pearson's correlations (N = 215).

		**M**	**SD**	**1**	**2**	**3**	**4**
1	TP	4.04	0.93	1			
2	SP	3.68	0.79	0.759^**^	1		
3	SoC	3.78	0.72	0.590^**^	0.572^**^	1	
4	CP	3.73	0.88	0.745^**^	0.759^**^	0.625^**^	1

TP means teaching presence, SP means social presence, SoC means sense of community, CP means cognitive presence.

** p < 0.01.

### Linear regression in the hypothesized model

**Figure 1:**
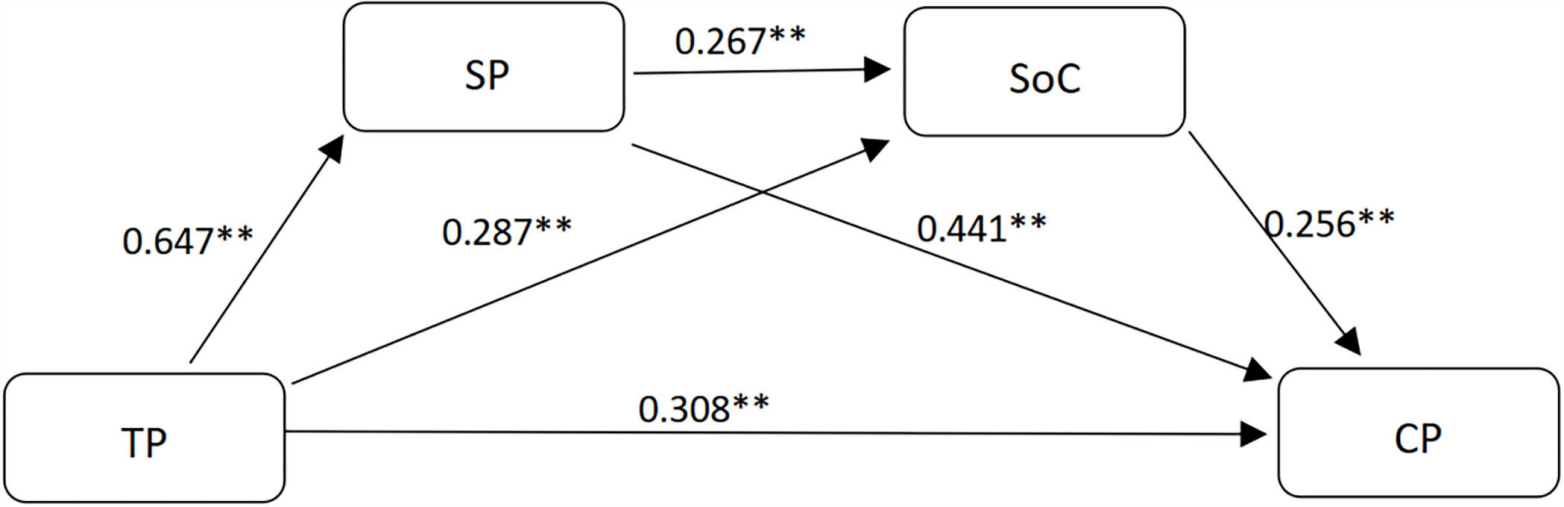
The mediation model. TP means teaching presence, SP means social presence, SoC means sense of community, CP means cognitive presence. **p < 0.001.

In this study, the direct pathways were examined by a simple linear regression analysis among the four variables. Results (see in Table 2; Figure 1) are from the simple linear regression analysis to test hypotheses 1 to 3, hypotheses 5 and 6, and hypothesis 8. The results indicate that TP is positively and significant correlated with SP (β = 0.647, *p* < 0.01), TP is positively correlated with SoC (β = 0.287, *p* < 0.01), SP is positively associated with SoC (β = 0.267, *p* < 0.01), TP has a positive and significant association with CP (β = 0.308, *p* < 0.01), SP is positively related to CP (β = 0.441, *p* < 0.01), and SoC is positively related to CP (β = 0.256, *p* < 0.01). The results concur with hypotheses 1 to 3, hypotheses 5 and 6, and hypothesis 8. Moreover, it echoes the study (Garrison et al., 2010) about the predicting roles of TP, SP, and SoC to CP.

**Table 2:** Results of simple linear regression.

**DV**	**IV**	**R**	**R2**	**β**	**t**
SP	TP	0.759	0.577	0.647	17.032^**^
SoC	TP	0.620	0.385	0.287	4.453^**^
	SP			0.267	3.525^**^
CP	TP	0.819	0.670	0.308	5.080^**^
	SP			0.441	6.301^**^
	SoC			0.256	4.138^**^

TP means teaching presence, SP means social presence, SoC means sense of community, CP means cognitive presence.

** p < 0.01.

There was a positive and statistically significant association between the independent variable and the dependent variable, therefore, path analysis was utilized to conduct the mediation analysis. The 5,000 re-sample bootstrapping method was used as the sample in this study and was not large enough to generalize the findings to all students in and beyond China. PROCESS v3.3 for SPSS was used to run the test and the direct effect is significant (β = 0.308, SE = 0.061, *t* = 5.0801, LLCI = 0.1885, ULCI = 0.4275, *p* < 0.001).

SP partially mediates the association between TP and CP (see Table 3). The indirect effect from TP to CP was β = 0.285 with the stand error (SE) of 0.052 (95% CI, 0.186 LLCI & 0.388 ULCI). The findings have supported our hypothesis 4 (H4)—SP mediates the relationship between TP and CP in blended learning during COVID-19. The mediating effect of SP alone was (β = 0.285, SE = 0.052), explaining 40.2% of the total effect. In addition, after including another mediator (SoC), the indirect effect from TP to CP was β = 0.073 with the stand error (SE) of 0.031 (95% CI, 0.020 LLCI & 0.140 ULCI). The results have also validated our hypothesis 7 (H7) assuming SoC to play a mediating role in the association between TP and CP in blended learning during COVID-19. The mediating effect of SoC alone was (β = 0.073, SE = 0.31), leading to 10.3% of the total effect.

**Table 3:** Testing the pathways of the mediation model (N = 215).

	**Effect**	**BootSE**	**BootLLCI**	**BootULCI**	**%**
Total ind	0.403	0.058	0.288	0.519	56.7
Ind1 TP → SP → CP	0.285	0.052	0.186	0.388	40.2
Ind2 TP → SoC → CP	0.073	0.031	0.020	0.140	10.3
Ind3 TP → SP → SoC → CP	0.044	0.020	0.011	0.089	6.2

TP means teaching presence, SP means social presence, SoC means sense of community, CP means cognitive presence. LLCI, lower limit of the confidence interval; ULCI, upper limit of the confidence interval.

Bootstrap sample size = 5,000.

At last, when including two mediators (SP and SoC), the indirect effect from TP to CP was decreased to β = 0.044 with the stand error (SE) of 0.020 (95% CI, 0.011 LLCI and 0.089 ULCI). Overall, the test results showed that students' perceived SP and SoC jointly played a chain mediating role between TP and CP. The total mediating effect of the two was (β = 0.403, SE = 0.058), accounting for 56.7% of the total effect. The chain mediating effect of SP and SoC was (β = 0.044, SE = 0.20), resulting in 6.2% of the total effect, thus hypothesis 9 (H9) has been verified.

## Discussion

### TP, SP, SoC, and CP

As indicated above, the rates of students' perceptions of TP, SP, SoC, and CP were described. In comparison with the results before COVID-19, results in this study showed a relatively higher mean score for TP (M = 4.04, SD = 0.93) in comparison with some studies involving different participants in distinct contexts before COVID-19, like online students from various kinds of learning institutions (Shea et al., 2006), undergraduate students in blended learning across disciplines (Ma et al., 2017), adult students in online/blended learning (Akyol et al., 2009), and online graduate students (Kozan, 2016), conducted among different disciplines including teacher leadership, management, education technology, and engineering. However, studies (Kozan and Richardson, 2014; Akyol and Garrison, 2019) showed a higher mean score of TP.

Students' perception of SP during the pandemic (M = 3.68, SD = 0.79) was in line with another study (Ma et al., 2017), which indicated a mean score of the same level (M = 3.61, SD = 0.53) in the Chinese context. However, students' perceptions of SP were generally lower than that of prior studies (Kozan and Richardson, 2014; Akyol and Garrison, 2019) with mean scores of more than 3.94 for SP. Nevertheless, some studies reported a mean score lower than 3.0 (Weidlich and Bastiaens, 2019) among German undergraduate students in education science and psychology. The context-specific nature of the three presences may help explain the variances in students' perceptions (Szeto, 2015).

Coming down to SoC, this study indicates higher SoC (M = 3.78, SD = 0.72) among students during the pandemic compared to studies that were conducted before the pandemic (Rovai, 2002a; Baturay and Bay, 2010; Nistor et al., 2015; YIlmaz, 2016; Shea, 2019). The literature showed that students in blended learning were more prone to establish a higher level of SoC (Rovai and Jordan, 2004). In addition, Chinese technological support and parental involvement may contribute to students' higher level of SoC during the COVID-19 period (Li et al., 2021).

Pertaining to the outcome variable, the mean score of students' CP during the pandemic (M = 3.73, SD = 0.88) was lower than that of previous studies (Akyol and Garrison, 2011; Kozan and Richardson, 2014), which were more than 4.0 in both online and blended context. However, other studies resulted in lower mean scores in the Chinese context even before the pandemic (Ma et al., 2017).

### The influence of TP, SP, and SoC on CP

Before running the mediation analysis, it was confirmed in the data preparation stage that students' perception of TP had a significant positive correlation with CP in blended learning during the pandemic, which supported hypothesis 1 (H1). With the result being statistically significant and positive (β = 0.308, *p* < 0.01), the direct association between students' perception of TP and CP was confirmed by the simple linear regression. It suggested that students who highly rated TP in blended learning during the pandemic felt a higher level of CP. Although similar studies were conducted in the K-12 context (Li et al., 2021) and sub-degree environment (Lau et al., 2021) in China during the pandemic, this study, to our best knowledge, might be the first one considering the higher education context of language learners in blended learning during the COVID-19 period. In accordance with findings of research conducted before COVID-19 (Akyol and Garrison, 2008; Ma et al., 2017; Zhao and Sullivan, 2017), TP was positively correlated with CP. As for peer interaction, some studies (Zhao and Sullivan, 2017; Wang and Liu, 2020) suggest that too much TP may not be conducive to students' participation in the interactive posting process and hamper students' knowledge assimilation, which might be due to the overwhelming centrality of TP, especially too much direct instruction (Wang and Liu, 2020). However, in this study, students find TP beneficial in guiding the learning process due to the pandemic.

With regard to hypothesis 2 (H2), it was predicted that students' perceived TP was positively and significantly related to their perceived SP. The results supported the hypothesis and confirmed the positive and significant association between students' perceived TP and SP (β = 0.647, *p* < 0.01), which was in line with most studies conducted before the pandemic (Garrison et al., 2010; Joo et al., 2011; d'Alessio et al., 2019). But Borup et al. (2015) asserted that TP (in specific indicator of feedback support of students' learning) had no significant impact on SP. The reason might be that video feedback in blended learning offsets the influence of the instructor SP. Yet, the central position of TP in the educational community of inquiry was strengthened by many studies (Hilliard and Stewart, 2019; Wang and Liu, 2020; Li et al., 2021), and so did the association between TP and SP.

Regarding the relationship between SP and CP of hypothesis 3 (H3), the study validated that students' perceived SP was positively and significantly correlated with students' perceived CP (β = 0.441, *p* < 0.01), as evidenced in online discussion forums where students were presented with complex cognitive tasks (Tirado Morueta et al., 2016). The association between SP and CP was moderately positive, which was in line with the results (β = 0.47, *p* < 0.01) of Archibald (2010), but was lower than that of (Kozan and Richardson, 2014). Studies with other operationalized measurements of SP and CP also demonstrated a statistically significant correlation (Ke, 2010; Gutiérrez-Santiuste et al., 2015).

Coming down to hypothesis 5 (H5), this study indicated that students' perceived TP was positively and significantly related to students' perceived SoC in blended learning during the pandemic (β = 0.287, *p* < 0.01). Related literature (Shea et al., 2006; Ice et al., 2007; Chen and Chiou, 2014; Wang and Liu, 2020) substantiated the positive and significant relationship between students' perceived TP and SoC. Other studies did not use the conception of TP but confirmed that course design and teacher facilitation promoted SoC (Oliphant and Branch-Mueller, 2016; Yapici, 2016).

Concerning the relationship between SoC and CP (hypothesis 6), the results in this study implied that a slightly significant correlation existed between students' perceived SoC and CP (β = 0.256, *p* < 0.0). Previous research demonstrated a positive correlation between SoC and academic achievement using standardized measures (Wighting et al., 2009), and also a positive relationship between SoC and perceived learning (Trespalacios and Perkins, 2016), the results of this study add to the existing literature that SoC is positively and significantly correlated with CP, extending the notion held by Akyol and Garrison (2011) that CP was closely related with students' perceived and actual learning outcomes (Joksimović et al., 2015; Rockinson-Szapkiw et al., 2016).

Hypothesis 8 (H8) assumed a positive and significant association between SP and SoC in blended learning during the COVID-19 Pandemic. Results confirmed that students' perceived SP was positively and significantly related to students' perceived SoC (β = 0.267, *p* < 0.01). Before the outbreak of COVID-19, studies (Tu and McIsaac, 2002; Lomicka and Lord, 2007; Remesal and Colomina, 2013; Chen and Chiou, 2014) confirmed the positive relationship between SP and SoC. It is believed that SP has a positive relationship with the sense of cohesion and with social capital (Wang et al., 2021).

### The mediating role of SP and SoC

The results showed that students' perception of TP had a direct positive influence on CP and had an indirect positive effect through the chain mediation of SP and SoC. The indirect effect paths were as follows: (1) TP → SP → CP (hypothesis 4), (2) TP → SoC → CP (hypothesis 7), and (3) TP → SP → SoC → CP (hypothesis 9).

First, SP positively affected CP and played a partial mediating role between TP and CP. The findings corroborated those of Shea and Bidjerano (2009), Garrison et al. (2010), Kozan and Richardson (2014), Ma et al. (2017) and Dempsey and Zhang (2019) conducted before the pandemic. However, the mediation role of SP in the relationship between TP and CP in the current study presents a higher impact than the previous study conducted before COVID-19 (Garrison et al., 2010). Literature showed that SP could predict learners' knowledge-sharing behavior in online learning communities (Karaoglan Yilmaz, 2017), as the pandemic pushed learning online or in blended forms, and it is understandable for SP to gain an increasing importance in promoting effective learning.

Second, SoC had a direct impact on CP and played a partial mediating role between TP and CP. The results were partially in line with research (Wighting et al., 2009; Trespalacios and Perkins, 2016; d'Alessio et al., 2019) conducted before the outbreak of COVID-19. Although some research validated the positive relationship between TP and SoC (Ice et al., 2007; d'Alessio et al., 2019), some other research related SoC with cognitive learning closely (Wighting et al., 2009; Trespalacios and Perkins, 2016), and there was a lack of empirical studies confirming the mediating role of SoC between TP and CP under the CoI framework. Moreover, Shea et al. (2014) called for more research about learners' SoC in online contexts through learners' recognition of connectedness, cohesion, and common learning goals rather than assessing learning itself; the study echoed this call in the pandemic times.

Third, SP and SoC acted as a complex chain mediating role in the relationship between TP and CP, accounting for 56.7% of the total effect, explaining more than half of the variance. This might be an important contribution of the current study, revealing a part of the complex relationship between TP and CP in blended learning during the COVID-19 pandemic. While previous studies have examined the mediating role of SP between TP and CP, little attention was paid to the possible mediation effect of SoC as well as the chain mediation roles of SP and SoC. As blended learning might become a new normal (Dziuban et al., 2018) in pandemic times, it is essential to fully understand learners' feelings of cohesion and connectedness based on socio-cognitive education philosophy. In contrast to Overbaugh and Lin (2006), who assumed some learners in a certain style might not need an SoC, we believed that SoC was vital for successful online or blended learning. Our findings showed that SP and SoC could strengthen the establishment of a blended learning community during the pandemic period.

## Limitations and implications for future studies

### Limitations

The results of the study were obtained during the COVID-19 period when the whole world suffered tremendously, and so did the field of higher education. In such a case, the possibility of signifying the significance of TP, SP, and SoC in CP by making the comparison with the findings gained in the period before or after the pandemic would be lessened. Samples from another setting or from another period may yield varying, yet distinct, findings.

Another limitation lay in the research context as samples came from just one university, though from diverse classes. As COVID-19 caused varying levels of disastrous impact in different countries, so did the impact on higher education, especially the institutional support of blended learning and student acceptance of new teaching modes in general; we should be careful to generalize this study results. Samples of the course College English might also not be generalizable enough as subjective feelings or perceptions might be discipline-specific. Although we take learners with different instructors into consideration to increase the sample diversity, the cross-sectional research design may bias causality results. It could be better to conduct a longitude design to investigate possible causal relationships.

### Implications for future studies

First, the time frame could be extended by future studies. When COVID-19 ends, research might investigate the correlations among TP, SP, SoC, and CP, and also examine the effect of TP, SP, and SoC on CP by using a different sample population (e.g., secondary school students), or the same sample, if possible. Nevertheless, we still emphasize that students' perceptions of TP, SP, and SoC are important antecedents of CP in blended language learning during COVID-19.

Second, future studies may take participants in different courses or samples from different cultural contexts into consideration to examine whether the effect of TP, SP, and SoC on TP is discipline dependent or culturally dependent. More participants from China should also be encouraged in future studies.

Finally, future research might adopt a qualitative method (e.g., interviews, reflective journals) or a mixed method to gain a deeper investigation of learners' perception of different presences in a CoI-based learning community. In addition, different potential predictors or antecedents of effective learning (including social capital, social interaction, technology acceptance, digital support, and knowledge sharing, etc.) could be taken into account in subsequent relevant studies.

## Conclusion

Given the findings of this study, the conclusion is that EFL learners' positive perceptions of TP and SP that promote their SoC carry the potential to promote their perceived CP in a CoI-based blended learning community during the COVID-19 period. The results might be of value to EFL educational researchers, practitioners, and instructors, who can recognize the importance of learners' socio-affective feelings/emotions in their learning process and take appropriate actions to promote effective learning. Increased CP is likely to have a positive effect on students' academic outcomes, such as deep learning and critical thinking capability (Garrison and Akyol, 2013). Therefore, on a practical level, behaviors fostering a positive blended learning climate (e.g., promoting pedagogical design, facilitation, and direct instruction—TP, affective expression, open communication, and group cohesion—SP, etc.) should be given due importance to teacher education. This study also stresses the socio-affective turn of academic research in blended learning and the theoretical framework of the CoI model that allows the current study to test the relationship among different presences and other possible variables.

## Data availability statement

The raw data supporting the conclusions of this article are available on reasonable request to the corresponding author.

## Ethics statement

Ethical review and approval was not required for the study on human participants in accordance with the local legislation and institutional requirements. The participants provided their written informed consent to participate in this study.

## Author contributions

LL: conceptualized, designed and drafted the manuscript, and submitted to the final version.

## Funding

This work was funded by Humanities and Social Sciences Research Project of the Ministry of Education of the People's Republic of China (Grant No. 21YJC740023).

## Conflict of interest

The author declares that the research was conducted in the absence of any commercial or financial relationships that could be construed as a potential conflict of interest.

## Publisher's note

All claims expressed in this article are solely those of the authors and do not necessarily represent those of their affiliated organizations, or those of the publisher, the editors and the reviewers. Any product that may be evaluated in this article, or claim that may be made by its manufacturer, is not guaranteed or endorsed by the publisher.
